# Cubic Vague Set and its Application in Decision Making

**DOI:** 10.3390/e22090963

**Published:** 2020-08-31

**Authors:** Khaleed Alhazaymeh, Yousef Al-Qudah, Nasruddin Hassan, Abdul Muhaimin Nasruddin

**Affiliations:** 1 Department of Basic Sciences and Mathematics, Faculty of Science, Philadelphia University, Amman 19392, Jordan; 2 Department of Mathematics, Faculty of Arts and Science, Amman Arab University, Amman 11953, Jordan; alquyousef82@gmail.com; 3 School of Mathematical Sciences, Faculty of Science and Technology, Universiti Kebangsaan Malaysia, Bangi 43600, Malaysia; nas@ukm.edu.my; 4 Department of Management and Marketing, School of Business and Economics, Universiti Putra Malaysia, Serdang 43400, Malaysia; abdulmuhaimin085@gmail.com

**Keywords:** cubic set, external cubic, fuzzy set, internal cubic, interval-valued, periodic, similarity measure, vague set

## Abstract

From the hybrid nature of cubic sets, we develop a new generalized hybrid structure of cubic sets known as cubic vague sets (CVSs). We also define the concept of internal cubic vague sets (ICVSs) and external cubic vague sets (ECVSs) with examples and discuss their interesting properties, including ICVSs and ECVSs under both P and R-Order. Moreover, we prove that the R and R-intersection of ICVSs (or ECVSs) need not be an ICVS (or ECVS). We also derive the different conditions for P-union (P-intersection, R and R-intersection) operations of both ICVSs (ECVSs) to become an ICVS (ECVS). Finally, we introduce a decision-making based on the proposed similarity measure of the CVSs domain and a numerical example is given to elucidate that the proposed similarity measure of CVSs is an important concept for measuring entropy in the information/data. It will be shown that the cubic vague set has the novelty to accurately represent and model two-dimensional information for real-life phenomena that are periodic in nature.

## 1. Introduction

In order to transact with several complicated problems involving uncertainties in many fields such as engineering, economics, social and medical sciences, classical methods are found to be inadequate. In 1965, Zadeh [1] presented fuzzy sets which helped to handle uncertainty and imprecision. Fuzzy sets had since been applied in many directions especially in decision making such as multi fuzzy sets [2], complex multi fuzzy sets [3,4,5,6,7], vague soft set [8,9,10,11], multiparameterized soft set [12], multi Q-fuzzy soft matrix [13] and intuitionistic fuzzy sets [14].

In fuzzy set theory, the grade of membership of an object to a fuzzy set indicates the belongingness degree of the object to the fuzzy set, which is a point (single) value selected from the unit interval [0, 1]. In real life scenarios, a person may consider that an element belongs to a fuzzy set, but it is possible that person is not sure about it. Therefore, hesitation or uncertainty may exist in which the element can belong to the fuzzy set or not. The traditional fuzzy set is unable to capture this type of hesitation or uncertainty using only the single membership degrees. A possible solution is to use an intuitionistic fuzzy set [14] or a vague set [15] to handle this problem. The vague set [15] is an extension of fuzzy sets and regarded as a special case of context-dependent fuzzy set which has the ability to overcome the problems faced when using fuzzy sets by providing us with an interval-based membership which clearly separates the evidence for and against an element.

From the above existing literature, we can see that those studies mainly focus on the fuzzy set, interval fuzzy set, vague set and their entropies [16,17,18]. Later on, Jun et al. [19] gave the idea of cubic set and it was characterized by interval valued fuzzy set and fuzzy set, which is a more general tool to capture uncertainty and vagueness, since fuzzy set deals with single-value membership while interval valued fuzzy set ranges the membership in the form of intervals. They presented the ideas of internal and external cubic sets and their characteristics. The hybrid platform provided by a cubic set has the main advantage since it contains more information than a fuzzy set and an interval-valued fuzzy set. By using this concept, different problems arising in several areas can be solved by means of cubic sets as in the works of Rashid et al. [20], Ma et al. [21], Khan et al. [22], Jun et al. [23,24], Gulistan et al. [25], Khaleed et al. [26], Fu et al. [27] and Ashraf et al. [28].

As for the Pythagorean fuzzy set (PFS) and its generalizations, an entropy measure was defined by Yang and Hussein [29]. Thao and Smarandache [30] proposed a new entropy measure for Pythagorean fuzzy which discarded the use of natural logarithm, while Wang and Li [31] introduced Pythagorean fuzzy interaction power Bonferroni mean aggregation operators in multiple attribute decision making.

Vague sets have a more powerful ability than fuzzy sets to process fuzzy information to some degree. Human cognition is usually a gradual process. As a result, how to characterize a vague concept and further measure its uncertainty becomes an interesting issue worth studying. Nevertheless, the concept of simple vague set is insufficient to provide the information about the occurrence of ratings or grades with accuracy because information is limited, and it is also unable to describe the occurrence of uncertainty and vagueness well enough, when sensitive cases are involved in decision making problems. Hence, there is a pertinent need for us to introduce the novel concept of cubic vague set (CVS) by incorporating both the ideas of cubic set and vague set. The aim of this model to introduce the notion of cubic vague set by extending the range of the truth-membership function and the false-membership function from a subinterval of [0, 1] to the interval-based membership structure that allows users to record their hesitancy in assigning membership values. This feature and its ability to represent two-dimensional information makes it ideal to be used to handle uncertain and subjective information that are prevalent in most time-periodic phenomena in the real world. These reasons served as the motivation to choose the cubic vague set model and use it in decision making problem.

The contribution of the novel cubic vague set (CVS) in the decision making process is its ability to handle uncertainties, imprecise and vagueness information considering both the truth-membership and falsity-membership values, whereas cubic set can only process the uncertainties information without able to take into account the truth-membership and falsity-membership values. The core advantage of using CVS against CS will be illustrated by an example. Hence, this concept of cubic vague set (CVS) will further enrich the use of various fuzzy methods in decision making such as those current trends which include group decision making using complex q-rung orthopair fuzzy Bonferroni mean [32], air pollution model using neutrosophic cubic Einstein averaging operators [33] and medicine preparation using neutrosophic bipolar fuzzy set [34].

The flow of our research is as follows. Firstly, we examine the concept of cubic vague set (CVS), which is a hybrid of vague set and cubic set. Secondly, we define some concepts related to the notion of CVS as well as some basic operations namely internal cubic vague sets (ICVSs) and external cubic vague sets (ECVSs). The CVS will be used together with a generalized algorithm to determine the similarity measures between two CVSs for a pattern recognition problem. Finally, a numerical example is given to elucidate that the proposed similarity measure of CVS is an important concept for measuring the entropy of uncertain information.

The organization of the paper will be as follows. Fundamentals of vague set, cubic set and interval-valued vague set are presented in Section 2. In Section 3, the concept of a cubic vague set with P- and R-union and P- and R-intersection for CVSs, with various properties are introduced. In Section 4, the similarity measure between CVSs is shown, along with an illustrative example studied, followed by the conclusion in Section 5.

## 2. Preliminaries

In this section we now state certain useful definitions, properties and several existing results for vague sets and cubic sets that will be useful for our discussion in the next sections.

The notion of vague set theory was first introduced by Gau and Buehrer in 1993 [15] as an extension of fuzzy sets. It is an improvement to deal with the vagueness of problems involving complex data with a high level of uncertainty and imprecision. Some of the basic concepts are as follows:

**Definition** **1.** *(See [15]) A vague set A (VS) in the universe of discourse U is a characterized by two membership functions given by:*

*1.*  *A truth membership function*
  $$t_{A}:U\to \left[0,1\right]$$
  *and*
*2.*  *a false membership function*
  $$f_{A}:U\to \left[0,1\right]$$
  *where $t_{A}\left(u\right)$ is a lower bound of the grade of membership of u derived from the “evidence for u", and $f_{A}\left(u\right)$ is a lower bound of the negation of u derived from the “evidence against u" and*
  $$t_{A}\left(u\right)+f_{A}\left(u\right)\leq 1.$$

*Thus, the grade of membership of u in the vague set A is bounded by a sub interval $\left[t_{A}\left(u\right),1-f_{A}\left(u\right)\right]$ of [0,1]. This indicates that if the actual grade of membership is μ(u), then*

$$t_{A}\left(u\right)\leq \mu \left(u\right)\leq 1-f_{A}\left(u\right).$$

*The vague set A is written as*

$$A=\{\left(u,\left[t_{A}\left(u\right),1-f_{A}\left(u\right)\right]\right)|u\in U\},$$

*where the interval $\left[t_{A}\left(u\right),1-f_{A}\left(u\right)\right]$ is called “vague value" of u in A and denoted by $V_{A}\left(u\right)$.*

**Definition** **2.** *(See [15]) The complement of a vague set A is denoted by $A^{c}$ and is defined by $t_{A^{c}}=f_{A},$ and $1-f_{A^{c}}=1-t_{A}.$*

**Definition** **3.** *(See [15]) The intersection of two VSs A and B are a VS C, written as C=A∩B, whose truth and false-membership functions for A and B by $t_{C}=\min\left(t_{A},t_{B}\right),$ and $1-f_{C}=\min\left(1-f_{A},1-f_{B}\right)=1-\max\left(f_{A},f_{B}\right).$*

**Definition** **4.** *(See [15]) The union of two VSs A and B are a VS C, written as C=A∪B, whose truth and false-membership for A and B by $t_{C}=\max\left(t_{A},t(B\right),$ and $1-f_{C}=\max\left(1-f_{A},1-f_{B}\right)=1-\min\left(f_{A},f_{B}\right).$*

Jun et al. [19] introduced the concept of a cubic set, as a novel hybrid structure of an interval-valued fuzzy set (IVFS) and a fuzzy set.

**Definition** **5.** *(See [19]) Let X be a non-empty set. A structure*

A={〈x,A(x),λ(x)〉|x∈X}

*be a cubic set in X in which A is an IVFS and λ is a fuzzy set in X.*

We will now introduce the concept of the interval-valued vague set to handle uncertainty of information, the grade of membership and the negation of *x*.

**Definition** **6.** *(See [15]) Let X be the universe of discourse, I[0,1] denotes the set of all closed subintervals of [0,1]. An interval valued vague set $A_{V}$ (IVVS) in X is a structure*

$$A_{V}=\left\{\langle x,t_{A_{V}}\left(x\right),f_{A_{V}}\left(x\right)\rangle :x\in X\right\}$$

*where $t_{A_{V}}:X\to I\left[0,1\right]$ and $f_{A_{V}}:X\to I\left[0,1\right]$ are truth-membership function and false-membership function of x concerning $A_{V}$, respectively. $t_{A_{V}}\left(x\right)=\left[t_{A_{V}}^{-}\left(x\right),t_{A_{V}}^{+}\left(x\right)\right]$, $t_{A_{V}}^{-}\left(x\right)$ and $t_{A_{V}}^{+}\left(x\right)$ denote the lower and upper bound of the grade of membership of x derived from “the evidence for x”, respectively. Similarly, $f_{A_{V}}\left(x\right)=\left[f_{A_{V}}^{-}\left(x\right),f_{A_{V}}^{+}\left(x\right)\right]$, $f_{A_{V}}^{-}\left(x\right)$ and $f_{A_{V}}^{+}\left(x\right)$ denote, respectively, the lower and upper bound of the negation of x derived from “the evidence against x”, and $t_{A_{V}}^{+}\left(x\right)+f_{A_{V}}^{+}\left(x\right)\leq 1$.*

## 3. Cubic Vague Sets

In this section, we will define the concept of a cubic vague set (CVS) and internal/external cubic vague sets.

**Definition** **7.** *Let X be a universal set. A cubic vague set $A^{V}$ defined over the universal set X is an ordered pair which is defined as follows*

$$A^{V}=\left\{\langle x,A_{V}\left(x\right),\lambda_{V}\left(x\right)\rangle :x\in X\right\}$$

*where $A_{V}=\langle A_{V}^{t},A_{V}^{1-f}\rangle =\left\{\langle x,\left[t_{A_{V}}^{-}\left(x\right),t_{A_{V}}^{+}\left(x\right)\right],\left[1-f_{A_{V}}^{-}\left(x\right),1-f_{A_{V}}^{+}\left(x\right)\right]\rangle :x\in X\right\}$ represents IVVS defined on X while $\lambda_{V}=\left\{\left(x,t_{\lambda_{V}}\left(x\right),1-f_{\lambda_{V}}\left(x\right)\right):x\in X\right\}$ represents *VS* such that $t_{A_{V}}^{+}\left(x\right)+f_{A_{V}}^{+}\left(x\right)\leq 1$ and $t_{\lambda_{V}}\left(x\right)+f_{\lambda_{V}}\left(x\right)\leq 1$. For clarity, we denote the pairs as $A^{V}=\langle A_{V},\lambda_{V}\rangle$, where $A_{V}=\langle \left[t_{A_{V}}^{-},t_{A_{V}}^{+}\right],\left[1-f_{A_{V}}^{-},1-f_{A_{V}}^{+}\right]\rangle$ and $\lambda_{V}=\left(t_{\lambda_{V}},1-f_{\lambda_{V}}\right)$. $C_{V}^{X}$ denotes the sets of all cubic vague sets in X.*

**Example** **1.** *Let X={a,b} be a universe set. Suppose an IVVS $A_{V}$ in X is defined by*

$$A_{V}=\left\{\langle \left[0.1,0.3\right],\left[0.3,0.7\right]\rangle /a,\langle \left[0.3,0.4\right],\left[0.5,0.6\right]\rangle /b\right\}$$

*and a VS $\lambda_{V}$ is a set of X is defined by*

$$\lambda_{V}=\left\{\left(0.5,0.7\right)/a,\left(0.1,0.3\right)/b\right\}.$$

*Then the cubic vague set $A^{V}=<A_{V},\lambda_{V}>$ will have the tabular representation as in Table 1.*

**Definition** **8.** *Let X be a universal set and V be a non-empty vague set. A cubic vague set $A^{V}=<A_{V},\lambda_{V}>$ is called an internal cubic vague set (brief. ICVS) if $A_{V}^{-}\left(x\right)\leq \lambda_{V}\left(x\right)\leq A_{V}^{+}\left(x\right)$ for all x∈X.*

**Definition** **9.** *Let X be a universal set and V be a non-empty vague set. A cubic vague set $A^{V}=<A_{V},\lambda_{V}>$ is called an external cubic vague set (brief. ECVS) if $\lambda_{V}\left(x\right)\notin \left(A_{V}^{-}\left(x\right),A_{V}^{+}\left(x\right)\right)$ for all x∈X.*

**Remark** **1.** *Let X be a universal set and V be a non-empty set. A cubic vague set $A^{V}=<A_{V},\lambda_{V}>$ is said to be neither ICVS nor ECVS if $A_{V}^{-}\left(x\right)\leq \lambda_{V}\left(x\right)\leq A_{V}^{+}\left(x\right)$ and $\lambda_{V}\left(x\right)\notin \left(A_{V}^{-}\left(x\right),A_{V}^{+}\left(x\right)\right)$ for all x∈X.*

**Example** **2.** *Let $A^{V}=\left\{<\left(x\right),A_{V}\left(x\right),\lambda_{V}\left(x\right)>:x\in X\right\}$ be a cubic vague set in X. If $A_{V}\left(x\right)=\left[0.2,0.5\right],\left[0.1,0.6\right]$ and $\lambda_{V}\left(x\right)=\left[0.2,0.5\right]$ for all x∈X, then $A^{V}$ is an ICVS. If $A_{V}\left(x\right)=\left[0.3,0.4\right],\left[0.2,0.5\right]$ and $\lambda_{V}\left(x\right)=\left[0.8,0.8\right]$, ∀x∈X, then $A^{V}$ is an ECVS. If $A_{V}\left(x\right)=\left[0.3,0.7\right],\left[0.4,0.5\right]$ and $\lambda_{V}\left(x\right)=\left[0.4,0.8\right]$, ∀x∈X, then $A^{V}$ is not an ICVS or an ECVS.*

**Theorem** **1.** *Let $A^{V}=<A_{V},\lambda_{V}>$ is be a CVS in X which is not an ECVS. Then ∃x∈X s.t $\lambda_{V}\left(x\right)\in \left(A_{V}^{-}\left(x\right),A_{V}^{+}\left(x\right)\right)$.*

**Proof.**  Straightforward. □

**Theorem** **2.** *Let $A^{V}=<A_{V},\lambda_{V}>$ is a CVS in X. If $A^{V}$ is an ICVS and ECVS, then*

(∀x∈X)(λ(x)∈W(A)∪M(A))

*where*

$$W\left(A\right)=\left\{A_{V}^{+}\left(x\right)|x\in X\right\}\;\mathrm{and}\;M\left(A\right)=\left\{A_{V}^{-}\left(x\right)|x\in X\right\}.$$

**Proof.**  Suppose that $A^{V}$ is an ICVS and ECVS. By using Definition 8 and Definition 9, we get $A_{V}^{-}\left(x\right)\leq \lambda_{V}\left(x\right)\leq A_{V}^{+}\left(x\right)$ and $\lambda_{V}\left(x\right)\notin \left(A_{V}^{-}\left(x\right),A_{V}^{+}\left(x\right)\right)\forall x\in X$. Thus, $\lambda_{V}\left(x\right)=A_{V}^{-}\left(x\right)$ or $\lambda_{V}\left(x\right)=A_{V}^{+}\left(x\right)$, and so $\lambda_{V}\left(x\right)\in W\left(A\right)\cup M\left(A\right)$. □

**Definition** **10.** *Let $A^{V}=\left\{<\left(x\right),A_{V}\left(x\right),\lambda_{V}\left(x\right)>:x\in X,v\in V\right\}$ and $B^{V}=\left\{<\left(x\right),B_{V}\left(x\right),\nu_{V}\left(x\right)>:x\in X,v\in V\right\}$ be two cubic vague sets in X and V. Then we have*

*1.*  *(Equality) $A^{V}=B^{V}\Leftrightarrow A_{V}\left(x\right)=B_{V}\left(x\right)$ and $\lambda_{V}\left(x\right)=\nu_{V}\left(x\right)$.*
*2.*  *(P-order) $A^{V}\subseteq_{P}B^{V}\Leftrightarrow A_{V}\left(x\right)\subseteq B_{V}\left(x\right)$ and $\lambda_{V}\left(x\right)\subseteq \nu_{V}\left(x\right)$.*
*3.*  *(R-order) $A^{V}\subseteq_{R}B^{V}\Leftrightarrow A_{V}\left(x\right)\subseteq B_{V}\left(x\right)$ and $\lambda_{V}\left(x\right)⊇\nu_{V}\left(x\right)$.*

**Definition** **11.** *The complement of $A^{V}=\left\{<\left(x\right),A_{V}\left(x\right),\lambda_{V}\left(x\right)>:x\in X,v\in V\right\}$ is defined to be the cubic vague set ${\left(A^{V}\right)}^{c}=\left\{<\left(x\right),A_{V}^{c}\left(x\right),\lambda_{V}^{c}\left(x\right)>:x\in X,v\in V\right\}$, where ${\left(A^{V}\right)}^{c}\left(x\right)=\left[1-{\left(A^{V}\right)}^{+}\left(x\right),1-{\left(A^{V}\right)}^{-}\left(x\right)\right]$ and $\lambda_{V}^{c}\left(x\right)$ is the vague complement $t_{A^{c}}\left(x\right)=f_{A}\left(x\right)$ and $1-f_{A^{c}}\left(x\right)=1-t_{A}\left(x\right)$.*

**Definition** **12.** *Let $A^{V}=\left\{<\left(x\right),A_{V}\left(x\right),\lambda_{V}\left(x\right)>:x\in X,v\in V\right\}$ and $B^{V}=\left\{<\left(x\right),B_{V}\left(x\right),\nu_{V}\left(x\right)>:x\in X,v\in V\right\}$ be two cubic vague sets in X and V. Then we have*

*1.*  *$A^{V}\cup_{P}B^{V}=\left\{<\left(x\right),\sup\left(A_{V}\left(x\right),B_{V}\left(x\right)\right),\sup\left(\lambda_{V}\left(x\right),\mu_{V}\left(x\right)\right)>|x\in X,v\in V\right\}$ (P-union).*
*2.*  *$A^{V}\cap_{P}B^{V}=\left\{<\left(x\right),\inf\left(A_{V}\left(x\right),B_{V}\left(x\right)\right),\inf\left(\lambda_{V}\left(x\right),\mu_{V}\left(x\right)\right)>|x\in X,v\in V\right\}$ (P-intersection).*
*3.*  *$A^{V}\cup_{R}B^{V}=\left\{<\left(x\right),\sup\left(A_{V}\left(x\right),B_{V}\left(x\right)\right),\inf\left(\lambda_{V}\left(x\right),\mu_{V}\left(x\right)\right)>|x\in X,v\in V\right\}$ (R-union).*
*4.*  *$A^{V}\cap_{R}B^{V}=\left\{<\left(x\right),\inf\left(A_{V}\left(x\right),B_{V}\left(x\right)\right),\sup\left(\lambda_{V}\left(x\right),\mu_{V}\left(x\right)\right)>|x\in X,v\in V\right\}$ (R-intersection).*
*5.*  *$A^{V}\wedge_{P}B^{V}=\left\{<\min\left(A_{V}\left(x\right),B_{V}\left(x\right)\right),\min\left(\lambda_{V}\left(x\right),\mu_{V}\left(x\right)\right)>|x\in X,v\in V\right\}$ (P-AND).*
*6.*  *$A^{V}\vee_{P}B^{V}=\left\{<\max\left(A_{V}\left(x\right),B_{V}\left(x\right)\right),\max\left(\lambda_{V}\left(x\right),\mu_{V}\left(x\right)\right)>|x\in X,v\in V\right\}$ (P-OR).*
*7.*  *$A^{V}\wedge_{R}B^{V}=\left\{<\min\left(A_{V}\left(x\right),B_{V}\left(x\right)\right),\max\left(\lambda_{V}\left(x\right),\mu_{V}\left(x\right)\right)>|x\in X,v\in V\right\}$ (R-AND).*
*8.*  *$A^{V}\vee_{R}B^{V}=\left\{<\max\left(A_{V}\left(x\right),B_{V}\left(x\right)\right),\min\left(\lambda_{V}\left(x\right),\mu_{V}\left(x\right)\right)>|x\in X,v\in V\right\}$ (R-OR).*

**Theorem** **3.** *Let $A^{V}=<A_{V},\lambda_{V}>$ be a CVS in X. If $A^{V}$ is ICVS (resp. ECVS), then $A^{V}c$ is also an ICVS (resp. ECVS).*

**Proof.**  Since $A^{V}=<A_{V}\left(x\right),\lambda_{V}\left(x\right)>$ is also an ICVS (resp. ECVS) in *X*, we have $A_{V}^{-}\left(x\right)\leq \lambda_{V}\left(x\right)\leq A_{V}^{+}\left(x\right)$ (resp. $\lambda_{V}\left(x\right)\notin \left(A_{V}^{-}\left(x\right),A_{V}^{+}\left(x\right)\right)$) for all x∈X. That means

$$1-A_{V}^{+}\left(x\right)\leq \lambda_{V}\left(x\right)\leq 1-A_{V}^{-}\left(x\right)$$

(resp. $1-\lambda_{V}\left(x\right)\notin \left(1-A_{V}^{+}\left(x\right),1-A_{V}^{-}\left(x\right)\right)$). Thus,

$$A^{V}c=\left\{\langle \left(x\right),A_{V}^{c}\left(x\right),\lambda_{V}^{c}\left(x\right)\rangle :x\in X,v\in V\right\}$$

is an ICVS (resp. ECVS) in *X*. □

**Theorem** **4.** *Let $A_{i}^{V}=\langle A_{i}{}_{V},\lambda_{i}{}_{V}|i\in A\rangle$ be a group of ICVSs in X. Then the P-union and intersection of $A_{i}^{V}=\langle A_{i}{}_{V},\lambda_{i}{}_{V}|i\in A\rangle$ are ICVSs in X.*

**Proof.**  Since $A_{i}^{V}$ is an ICVS in *X*, we have $A_{i}{}_{V}^{-}\left(x\right)\leq \lambda_{i}{}_{V}\left(x\right)\leq A_{i}{}_{V}^{+}\left(x\right)$ for i∈A. That means

$${\left(\underset{i\in A}{⋃}A_{i}\right)}^{-}\left(x\right)\leq \left(\underset{i\in A}{⋁}\lambda_{i}{}_{V}\right)\left(x\right)\leq {\left(\underset{i\in A}{⋃}A_{i}\right)}^{+}\left(x\right)$$

and

$${\left(\underset{i\in A}{⋂}A_{i}\right)}^{-}\left(x\right)\leq \left(\underset{i\in A}{⋀}\lambda_{i}{}_{V}\right)\left(x,v\right)\leq {\left(\underset{i\in A}{⋂}A_{i}\right)}^{+}\left(x\right)$$

Hence $\underset{i\in A}{⋃}{}_{P}A_{i}^{V}$ and $\underset{i\in A}{⋂}{}_{P}A_{i}^{V}$ are ICVSs in *X*. □

The following example shows that the P-union and P-intersection of two ECVSs need not be an ECVS.

**Example** **3.** *Let $A^{V}=\langle A_{V}\left(x\right),\lambda_{V}\left(x\right)\rangle$ and $B^{V}=\langle B_{V}\left(x\right),\nu_{V}\left(x\right)\rangle$ be two ECVSs in X such that $A_{V}\left(x\right)=\left[0.3,0.4\right],\left[0.5,0.8\right]$ and λ(x)=[0.9,0.9], $B_{V}\left(x\right)=\left[0.8,0.8\right],\left[0.9,0.9\right]$ and ν(x)=[0.3,0.4]∀x∈X.*

*1.*  *Note that $A^{V}\cup_{P}B^{V}=\left\{\langle \left(x\right),B\left(x\right),\lambda \left(x\right)|x\in I\rangle \right\}$ and $\lambda \left(x\right)\in \left(B^{-}\left(x\right),B^{+}\left(x\right)\right)$∀x∈I. Then $A^{V}\cup_{P}B^{V}$ is not an ECVS in I.*
*2.*  *Note that $A^{V}\cap_{P}B^{V}=\left\{\langle \left(x\right),A\left(x\right),\nu \left(x\right)|x\in I\rangle \right\}$ and $\nu \left(x\right)\in \left(A^{-}\left(x\right),A^{+}\left(x\right)\right)$∀x∈I. Then $A^{V}\cap_{P}B^{V}$ is not an ECVS in I.*

The example below shows that the R-union and intersection of two ICVSs need not be an ICVS.

**Example** **4.** *Let $A^{V}=\langle A_{V}\left(x\right),\lambda_{V}\left(x\right)\rangle$ and $B^{V}=\langle B_{V}\left(x\right),\nu_{V}\left(x\right)\rangle$ be ICVSs in I=[0,1]×[0,1] in which $A_{V}\left(x\right)=\left[0.4,0.5\right],\left[0.4,0.7\right]$, λ(x)=[0.6,0.7], $B_{V}\left(x\right)=\left[0.7,0.9\right],\left[0.8,1\right]$ and ν(x)=[0.8,0.8] for all x∈I.*

*1.*  *Note that $A^{V}\cup_{R}B^{V}=\left\{\langle \left(x\right),B\left(x\right),\lambda \left(x\right)|x\in I\rangle \right\}$ and $\lambda \left(x\right)\notin \left(B^{-}\left(x\right),B^{+}\left(x\right)\right)\forall x\in I$. Then $A^{V}\cup_{R}B^{V}$ is not an ICVS in I.*
*2.*  *Note that $A^{V}\cap_{R}B^{V}=\left\{\langle \left(x\right),A\left(x\right),\nu \left(x\right)|x\in I\rangle \right\}$ and $\nu \left(x\right)\notin \left(A^{-}\left(x\right),A^{+}\left(x\right)\right)\forall x\in I$. Then $A^{V}\cap_{R}B^{V}$ is not an ICVS in I.*

The example below will show that the R-union and intersection of two ECVSs may not necessarily be an ECVS.

**Example** **5.**

*1.*  *Let $A^{V}=\langle A_{V}\left(x\right),\lambda_{V}\left(x\right)\rangle$ and $B^{V}=\langle B_{V}\left(x\right),\nu_{V}\left(x\right)\rangle$ be ECVSs in I=[0,1]×[0,1] whereas $A_{V}\left(x\right)=\left[0.4,0.7\right],\left[1,1\right]$, λ(x)=[0.8,0.9], $B_{V}\left(x\right)=\left[0.5,0.8\right],\left[0.6,0.9\right]$ and ν(x)=[0.4,1]∀x∈I. Since $A^{V}\cup_{R}B^{V}=\left\{\langle \left(x\right),B_{V}\left(x\right),\lambda \left(x\right)|x\in I\rangle \right\}$ and $\lambda \left(x\right)\in \left(B^{-}\left(x\right),B^{+}\left(x\right)\right)\forall x\in I$. Then $A^{V}\cup_{R}B^{V}$ is not an ECVS in I.*
*2.*  *Let $A^{V}=\langle A_{V}\left(x\right),\lambda_{V}\left(x\right)\rangle$ and $B^{V}=\langle B_{V}\left(x\right),\nu_{V}\left(x\right)\rangle$ be ECVSs in I=[0,1]×[0,1] whereas $A_{V}\left(x\right)=\left[0.5,0.7\right]$, λ(x)=[0.8,0.9], $B_{V}\left(x\right)=\left[0.2,0.3\right]$ and ν(x)=[0.6,0.6] for all x∈I. Since $A^{V}\cap_{R}B^{V}=\left\{\langle \left(x\right),A\left(x\right),\nu \left(x\right)|x\in I\rangle \right\}$ and $\nu \left(x\right)\in \left(A^{-}\left(x\right),A^{+}\left(x\right)\right)\forall x\in I$. Then $A^{V}\cap_{R}B^{V}$ is not an ECVS in I.*

We give a condition of a R-union of two ICVSs to become an ICVS.

**Theorem** **5.** *Let $A^{V}=\langle A_{V}\left(x\right),\lambda_{V}\left(x\right)\rangle$ and $B^{V}=\langle B_{V}\left(x\right),\nu_{V}\left(x\right)\rangle$ be two ICVSs in X such that*

$$\max\{A_{V}^{-}\left(x\right),B_{V}^{-}\left(x\right)\}\leq \left(\lambda \wedge \nu \right)\left(x\right)$$

∀x∈X. *Then the R-union of*$A^{V}$*and*$B^{V}$*is an ICVS in X.*

**Proof.**  Let $A^{V}=\langle A_{V}\left(x\right),\lambda_{V}\left(x\right)\rangle$ and $B^{V}=\langle B_{V}\left(x\right),\nu_{V}\left(x\right)\rangle$ be two ICVSs in *X* which satisfy the condition of Definition 7. Then $A_{V}^{-}\left(x\right)\leq \lambda_{V}\left(x\right)\leq A_{V}^{+}\left(x\right)$ and $B_{V}^{-}\left(x\right)\leq \nu_{V}\left(x\right)\leq B_{V}^{+}\left(x\right)$ which means $\left(\lambda_{V}\left(x\right)\wedge \nu_{V}\left(x\right)\right)\leq {\left(A\cup B\right)}^{+}\left(x\right)$. Now apply the condition of Definition 7 that is ${\left(A\cup B\right)}^{-}\left(x\right)=\max\left\{A_{V}^{-}\left(x\right),B_{V}^{-}\left(x\right)\right\}\leq \left(\lambda_{V}\left(x\right)\wedge \nu_{V}\left(x\right)\right)\leq {\left(A\cup B\right)}^{+}\left(x\right)$ so that $A^{V}\cup_{R}B^{V}=\left\{\langle \left(x\right),\left(A\cup B\right)\left(x\right),\left(\lambda \wedge \nu \right)\left(x\right)\rangle |x\in X\right\}$ is an ICVS in *X*. □

We give a condition of a R-intersection of two ICVSs to become an ICVS.

**Theorem** **6.** *Let $A^{V}=\langle A_{V}\left(x\right),\lambda_{V}\left(x\right)\rangle$ and $B^{V}=\langle B_{V}\left(x\right),\nu_{V}\left(x\right)\rangle$ be two ICVSs in X satisfying $\min\{A_{V}^{+}\left(x\right),B_{V}^{+}\left(x\right)\}\geq \left(\lambda \vee \nu \right)\left(x\right)\forall x\in X$. Then the R-intersection of $A^{V}$ and $B^{V}$ is an IVCS in X.*

**Proof.**  Let $A^{V}=\langle A_{V}\left(x\right),\lambda_{V}\left(x\right)\rangle$ and $B^{V}=\langle B_{V}\left(x\right),\nu_{V}\left(x\right)\rangle$ be ICVSs in *X* which satisfy the condition of Definition 1. Then $A_{V}^{-}\left(x\right)\leq \lambda_{V}\left(x\right)\leq A_{V}^{+}\left(x\right)$ and $B_{V}^{-}\left(x\right)\leq \nu_{V}\left(x\right)\leq B_{V}^{+}\left(x\right)$ so ${\left(A\cap B\right)}^{-}\left(x\right)\leq \left(\lambda_{V}\left(x\right)\wedge \nu_{V}\left(x\right)\right)$. Now apply the condition of Definition 1 we get ${\left(A\cap B\right)}^{-}\left(x\right)\leq \left(\lambda_{V}\left(x\right)\wedge \nu_{V}\left(x\right)\right)\leq \min\left\{A_{V}^{+}\left(x\right),B_{V}^{+}\left(x\right)\right\}={\left(A\cap B\right)}^{+}\left(x\right)$ and therefore $A^{V}\cap_{R}B^{V}=\left\{\langle \left(x\right),\left(A\cap B\right)\left(x\right),\left(\lambda \vee \nu \right)\left(x\right)\rangle |x\in X\right\}$ is an ICVS in *X*. □

Given two CVSs $A^{V}=\langle A_{V}\left(x\right),\lambda_{V}\left(x\right)\rangle$ and $B^{V}=\langle B_{V}\left(x\right),\nu_{V}\left(x\right)\rangle$. Suppose we exchange the ν for λ in the two CVSs and we denote the CVSs $A^{*}=\langle A_{V}\left(x\right),\nu_{V}\left(x\right)\rangle$ and $B^{*}=\langle B_{V}\left(x\right),\lambda_{V}\left(x\right)\rangle$, respectively. Then, for to ECVSs $A^{V}$ and $B^{V}$ in *X*, two cubic vague sets $A^{*}$ and $B^{*}$ may not be ICVSs in *X* as shown in the example below.

**Example** **6.**

*1.*  Let $A^{V}=\langle A_{V}\left(x\right),\lambda_{V}\left(x\right)\rangle$ and $B^{V}=\langle B_{V}\left(x\right),\nu_{V}\left(x\right)\rangle$ be two EVCSs in I=[0,1]×[0,1] in which A(x)=[0.2,0.2],[0.5,0.5], λ(x)=[0.3,0.4], B(x)=[0.7,0.8],[0.9,0.9] and ν(x)=[0.1,0.1]∀x∈I. Thus, $A^{*}=\langle A_{V}\left(x\right),\nu_{V}\left(x\right)\rangle$ and $B^{*}=\langle B_{V}\left(x\right),\lambda_{V}\left(x\right)\rangle$ are not ICVSs in X since $\nu_{V}\left(x\right)=\left[0.1,0.1\right]\notin \left[0.2,0.3\right],\left[0.5,0.5\right]$ and $\lambda_{V}\left(x\right)=\left[0.3,0.4\right]\notin \left[0.7,0.8\right],\left[0.9,0.9\right]$.
*2.*  Let X={l,m} be a set. Let $A^{V}=\langle A_{V}\left(x\right),\lambda_{V}\left(x\right)\rangle$ and $B^{V}=\langle B_{V}\left(x\right),\nu_{V}\left(x\right)\rangle$ be ECVSs in X defined by Table 2. Thus, $A^{*}=\langle A_{V}\left(x\right),\nu_{V}\left(x\right)\rangle$ and $B^{*}=\langle B_{V}\left(x\right),\lambda_{V}\left(x\right)\rangle$ are not ICVSs in X since ν(l)=[0.9,0.9]∉[0.2,0.5],[0.5,0.5]=A(l) and λ(l)=[0.1,0.7]∉[0.8,0.9],[0.8,0.8].

We give an example to show that the P-union of two ECVSs in *X* does not necessarily become an ICVS in *X*.

**Example** **7.** *Let X={k,l,m} be a set. Let $A^{V}=\langle A_{V}\left(x\right),\lambda_{V}\left(x\right)\rangle$ and $B^{V}=\langle B_{V}\left(x\right),\nu_{V}\left(x\right)\rangle$ be two ECVSs in X defined by Table 3. Then we will have $\left(A^{V}\cup_{P}B^{V}\right)=\langle \left(A_{V}\left(x\right)\cup B_{V}\left(x\right)\right),\left(\lambda_{V}\left(x\right)\vee \nu_{V}\left(x\right)\right)\rangle$ is not an ICVS in X since $\left(\lambda \vee \nu \right)\left(m\right)=\left[0.2,0.9\right]\notin \left(\left[0.1,0.1\right],\left[0.7,0.8\right]\right)$*

We give a condition for P-union of two ECVSs to become an ICVS.

**Theorem** **7.** For two ECVSs $A^{V}=\langle A_{V}\left(x\right),\lambda_{V}\left(x\right)\rangle$ and $B^{V}=\langle B_{V}\left(x\right),\nu_{V}\left(x\right)\rangle$ in X, if $A^{*}=\langle A_{V}\left(x\right),\nu_{V}\left(x\right)\rangle$ and $B^{*}=\langle B_{V}\left(x\right),\lambda_{V}\left(x\right)\rangle$ are IVCSs in X, then the P-union $A^{V}\cup_{P}B^{V}$ of $A^{V}=\langle A_{V}\left(x\right),\lambda_{V}\left(x\right)\rangle$ and $B^{V}=\langle B_{V}\left(x\right),\nu_{V}\left(x\right)\rangle$ is an ICVS in X.

**Proof.**  Let $A^{V}=\langle A_{V}\left(x\right),\lambda_{V}\left(x\right)\rangle$ and $B^{V}=\langle B_{V}\left(x\right),\nu_{V}\left(x\right)\rangle$ be an ECVSs in *X* such that $A^{*}=\langle A_{V}\left(x\right),\nu_{V}\left(x\right)\rangle$ and $B^{*}=\langle B_{V}\left(x\right),\lambda_{V}\left(x\right)\rangle$ are ICVSs in *X*. Then $\lambda_{V}\left(x\right)\notin \left(A_{V}^{-}\left(x\right),A_{V}^{+}\left(x\right)\right)$, $\mu_{V}\left(x\right)\notin \left(B_{V}^{-}\left(x\right),B_{V}^{+}\left(x\right)\right)$, $B_{V}^{-}\left(x\right)\leq \lambda_{V}\left(x\right)\leq B_{V}^{+}\left(x\right)$ and $A_{V}^{-}\left(x\right)\leq \mu_{V}\left(x\right)\leq A_{V}^{+}\left(x\right)$ for all x∈X. Now, for a given x∈X, we consider the cases:

1. $\lambda_{V}\left(x\right)\leq A_{V}^{-}\left(x\right)\leq \nu_{V}\left(x\right)\leq A_{V}^{+}\left(x\right)$ and $\nu_{V}\left(x\right)\leq B_{V}^{-}\left(x\right)\leq \lambda_{V}\left(x\right)\leq B_{V}^{+}\left(x\right)$.
2. $A_{V}^{-}\left(x\right)\leq \nu_{V}\left(x\right)\leq A_{V}^{+}\left(x\right)\leq \lambda_{V}\left(x\right)$ and $B_{V}^{-}\left(x\right)\leq \lambda_{V}\left(x\right)\leq B_{V}^{+}\left(x\right)\leq \nu_{V}\left(x\right)$.
3. $\lambda_{V}\left(x\right)\leq A_{V}^{-}\left(x\right)\leq \nu_{V}\left(x\right)\leq A_{V}^{+}\left(x\right)$ and $B_{V}^{-}\left(x\right)\leq \lambda_{V}\left(x\right)\leq B_{V}^{+}\left(x\right)\leq \nu_{V}\left(x\right)$.
4. $A_{V}^{-}\left(x\right)\leq \nu_{V}\left(x\right)\leq A_{V}^{+}\left(x\right)\leq \lambda_{V}\left(x\right)$ and $\nu_{V}\left(x\right)\leq B_{V}^{-}\left(x\right)\leq \lambda_{V}\left(x\right)\leq B_{V}^{+}\left(x\right)$.

We will illustrate the proof of the first case only because proofs of the remaining three cases are similar. Now, we get $\mu_{V}\left(x\right)=A_{V}^{-}\left(x\right)=B_{V}^{-}\left(x\right)=\lambda_{V}\left(x\right)$. Since $A^{*}=\langle A_{V}\left(x\right),\nu_{V}\left(x\right)\rangle$ and $B^{*}=\langle B_{V}\left(x\right),\lambda_{V}\left(x\right)\rangle$ are ICVSs in *X*, we have $\nu_{V}\left(x\right)\leq A_{V}^{+}\left(x\right)$ and $\lambda_{V}\left(x\right)\leq B_{V}^{+}\left(x\right)$. It follows that

(1) $$\begin{array}{c} {\left(A^{V}\cup B^{V}\right)}^{-}\left(x\right)=\max\left\{A_{V}^{-}\left(x\right),B_{V}^{-}\left(x\right)\right\}=\left(\lambda_{V}\left(x\right),\nu_{V}\left(x\right)\right)\\ \leq \max\left\{A_{V}^{+}\left(x\right),B_{V}^{+}\left(x\right)\right\}={\left(A^{V}\cup B^{V}\right)}^{+}\left(x\right) \end{array}$$

Hence $A^{V}\cup_{P}B^{V}$ is an ICVS in *X*. □

We give the condition of a P-intersection of two ECVSs to become an ICVS.

**Theorem** **8.** Let $A^{V}=\langle A_{V}\left(x\right),\lambda_{V}\left(x\right)\rangle$ and $B^{V}=\langle B_{V}\left(x\right),\nu_{V}\left(x\right)\rangle$ be CVSs in X such that $A^{*}=\langle A_{V}\left(x\right),\nu_{V}\left(x\right)\rangle$ and $B^{*}=\langle B_{V}\left(x\right),\lambda_{V}\left(x\right)\rangle$ are ICVSs in X. Then the P-intersection of $A^{V}$ and $B^{V}$ is an ICVS in X.

**Proof.**  The proof is similar to that of Theorem 7. □

For two ECVSs $A^{V}$ and $B^{V}$ in *X*, two CVSs $A^{*}=\langle A_{V}\left(x\right),\nu_{V}\left(x\right)\rangle$ and $B^{*}=\langle B_{V}\left(x\right),\lambda_{V}\left(x\right)\rangle$ may not be ECVSs as shown in the following example.

**Example** **8.** Let X={l,m} be a set. Let $A^{V}=\langle A_{V}\left(x\right),\lambda_{V}\left(x\right)\rangle$ and $B^{V}=\langle B_{V}\left(x\right),\nu_{V}\left(x\right)\rangle$ be ECVSs in X given in Table 4. Thus, $A^{*}=\langle A_{V}\left(x\right),\nu_{V}\left(x\right)\rangle$ and $B^{*}=\langle B_{V}\left(x\right),\lambda_{V}\left(x\right)\rangle$ are not ECVSs in X since ν(m)=[0.3,0.3]∈[0.3,0.3],[0.4,0.5]=A(m) and λ(l)=[0.6,0.7]∈[0.6,0.8],[0.7,0.8].

**Theorem** **9.** Let $A^{V}=\langle A_{V}\left(x\right),\lambda_{V}\left(x\right)\rangle$ and $B^{V}=\langle B_{V}\left(x\right),\nu_{V}\left(x\right)\rangle$ be two ECVSs in X such that $A^{*}=\langle A_{V}\left(x\right),\nu_{V}\left(x\right)\rangle$ and $B^{*}=\langle B_{V}\left(x\right),\lambda_{V}\left(x\right)\rangle$ are ECVSs in X. Thus, the P-union $A^{V}$ and $B^{V}$ is an ECVS in X.

**Proof.**  For each x∈X, we get $\lambda_{V}\left(x\right)\notin \left(A_{V}^{-}\left(x\right),A_{V}^{+}\left(x\right)\right)$, $\nu_{V}\left(x\right)\notin \left(B_{V}^{-}\left(x\right),B_{V}^{+}\left(x\right)\right)$, $\nu_{V}\left(x\right)\notin \left(A_{V}^{-}\left(x\right),A_{V}^{+}\left(x\right)\right)$ and $\lambda_{V}\left(x\right)\notin \left(B_{V}^{-}\left(x\right),B_{V}^{+}\left(x\right)\right)$

$$\left(\lambda \vee \nu \right)\left(x\right)\notin \left(\max\left\{A_{V}^{-}\left(x\right),B_{V}^{-}\left(x\right)\right\},\max\left\{A_{V}^{+}\left(x\right),B_{V}^{+}\left(x\right)\right\}\right)$$

which means $\left(\lambda \vee \nu \right)\left(x\right)\notin \left({\left(A\cup B\right)}^{-}\left(x\right),\left(A\cup B\right)\left(x\right)\right)$. Then $A^{V}\cup_{P}B^{V}$ is an ECVS in *X*. □

We have given an example that shows the P-intersection for two ECVSs may not become an ECVS as in Example 3. Now we will add a condition for the P-itersection of two ECVSs to be an ECVS by using Definition 2.

**Theorem** **10.** *Let $A^{V}=\langle A_{V}\left(x\right),\lambda_{V}\left(x\right)\rangle$ and $B^{V}=\langle B_{V}\left(x\right),\nu_{V}\left(x\right)\rangle$ be two ECVSs in X such that $\min\{\max\left\{A_{V}^{+}\left(x\right),B_{V}^{-}\left(x\right)\right\},\max\left\{A_{V}^{-}\left(x\right),B_{V}^{+}\left(x\right)\right\}\}$≥(λ∨ν)(x)$>\max\{\min\left\{A_{V}^{+}\left(x\right),B_{V}^{-}\left(x\right)\right\},\min\left\{A_{V}^{-}\left(x\right),B_{V}^{+}\left(x\right)\right\}\}\forall x\in X$. Thus, the P-intersection $A^{V}$ and $B^{V}$ is an ECVS in X.*

**Proof.**  For each x∈X, substitute

$$\psi_{\left(x\right)}:=\min\left\{\max\left\{A_{V}^{+}\left(x\right),B_{V}^{-}\left(x\right)\right\},\max\left\{A_{V}^{-}\left(x\right),B_{V}^{+}\left(x\right)\right\}\right\}$$

and

$$\phi_{\left(x\right)}:=\max\left\{\min\left\{A_{V}^{+}\left(x\right),B_{V}^{-}\left(x\right)\right\},\min\left\{A_{V}^{-}\left(x\right),B_{V}^{+}\left(x\right)\right\}\right\}$$

Then $\psi_{\left(x\right)}$ which is one of the $A_{V}^{-}\left(x\right),B_{V}^{-}\left(x\right),A_{V}^{+}\left(x\right)$ and $B_{V}^{+}\left(x\right)$. We consider $\psi_{\left(x\right)}=A_{V}^{-}\left(x\right)$ or $\psi_{\left(x\right)}=A_{V}^{+}\left(x\right)$ only since the proof of the other cases are similar.

If $\psi_{\left(x\right)}=A_{V}^{-}\left(x\right)$, thus

$$B_{V}^{-}\left(x\right)\leq B_{V}^{+}\left(x\right)\leq A_{V}^{-}\left(x\right)\leq A_{V}^{+}\left(x\right)$$

and so $\phi_{\left(x\right)}=B_{V}^{+}\left(x\right)$. Then $B_{V}^{-}\left(x\right)={\left(A\cap B\right)}^{-}\left(x\right)\leq {\left(A\cap B\right)}^{+}\left(x\right)=B_{V}^{+}\left(x\right)=\phi_{\left(x\right)}<\left(\lambda \wedge \nu \right)\left(x\right),$ thus $\left(\lambda \wedge \nu \right)\left(x\right)\notin \left({\left(A\cap B\right)}^{-}\left(x\right),{\left(A\cap B\right)}^{+}\left(x\right)\right)$.

If $\psi_{\left(x\right)}=A_{V}^{+}\left(x\right)$, thus $B_{V}^{-}\left(x\right)\leq A_{V}^{+}\left(x\right)\leq B_{V}^{+}\left(x\right)$ and so $\phi_{\left(x\right)}=\max\left\{A_{V}^{-}\left(x\right),B_{V}^{-}\left(x\right)\right\}$. Suppose that, $\phi_{\left(x\right)}=A_{V}^{-}\left(x\right)$. Thus, $B_{V}^{-}\left(x\right)\leq A_{V}^{-}\left(x\right)<\left(\lambda \wedge \nu \right)\left(x\right)\leq A_{V}^{+}\left(x\right)\leq B_{V}^{+}\left(x\right)$ then we get

$$B_{V}^{-}\left(x\right)\leq A_{V}^{-}\left(x\right)<\left(\lambda \wedge \nu \right)\left(x\right)<A_{V}^{+}\left(x\right)\leq B_{V}^{+}\left(x\right)$$

or

$$B_{V}^{-}\left(x\right)\leq A_{V}^{-}\left(x\right)<\left(\lambda \wedge \nu \right)\left(x\right)=A_{V}^{+}\left(x\right)\leq B_{V}^{+}\left(x\right)$$

of the case $B_{V}^{-}\left(x\right)\leq A_{V}^{-}\left(x\right)<\left(\lambda \wedge \nu \right)\left(x\right)<A_{V}^{+}\left(x\right)\leq B_{V}^{+}\left(x\right)$. This is the contradiction to $A^{V}$ and $B^{V}$ are ECVSs in *X*. For the case

$$B_{V}^{-}\left(x\right)\leq A_{V}^{-}\left(x\right)<\left(\lambda \wedge \nu \right)\left(x\right)=A_{V}^{+}\left(x\right)\leq B_{V}^{+}\left(x\right)$$

we get $\left(\lambda \wedge \nu \right)\left(x\right)\notin \left({\left(A\cap B\right)}^{-}\left(x\right),{\left(A\cap B\right)}^{+}\left(x\right)\right)$ because $\left(\lambda \wedge \nu \right)\left(x\right)=A_{V}^{+}\left(x\right)={\left(A\cap B\right)}^{+}\left(x\right)$.

Suppose that, $\phi_{\left(x\right)}=B_{V}^{-}\left(x\right)$. Thus,

$$A_{V}^{-}\left(x\right)\leq B_{V}^{-}\left(x\right)<\left(\lambda \wedge \nu \right)\left(x\right)\leq A_{V}^{+}\left(x\right)\leq B_{V}^{+}\left(x\right)$$

then we get

$$A_{V}^{-}\left(x\right)\leq B_{V}^{-}\left(x\right)<\left(\lambda \wedge \nu \right)\left(x\right)<A_{V}^{+}\left(x\right)\leq B_{V}^{+}\left(x\right)$$

or

$$A_{V}^{-}\left(x\right)\leq B_{V}^{-}\left(x\right)<\left(\lambda \wedge \nu \right)\left(x\right)=A_{V}^{+}\left(x\right)\leq B_{V}^{+}\left(x\right)$$

of the case $A_{V}^{-}\left(x\right)\leq B_{V}^{-}\left(x\right)<\left(\lambda \wedge \nu \right)\left(x\right)<A_{V}^{+}\left(x\right)\leq B_{V}^{+}\left(x\right)$. This is the contradiction to $A^{V}$ and $B^{V}$ are ECVSs in *X*. For the case

$$A_{V}^{-}\left(x\right)\leq B_{V}^{-}\left(x\right)<\left(\lambda \wedge \nu \right)\left(x\right)=A_{V}^{+}\left(x\right)\leq B_{V}^{+}\left(x\right)$$

we get $\left(\lambda \wedge \nu \right)\left(x\right)\notin \left({\left(A\cap B\right)}^{-}\left(x\right),{\left(A\cap B\right)}^{+}\left(x\right)\right)$ because $\left(\lambda \wedge \nu \right)\left(x\right)=A_{V}^{+}\left(x\right)={\left(A\cap B\right)}^{+}\left(x\right)$. Then P-intersection of $A^{V}$ and $B^{V}$ are ECVSs in *X*. □

We add a condition of a P-intersection of two CVSs to become both an ECVS and ICVS.

**Theorem** **11.** *Let $A^{V}=\langle A_{V}\left(x\right),\lambda_{V}\left(x\right)\rangle$ and $B^{V}=\langle B_{V}\left(x\right),\nu_{V}\left(x\right)\rangle$ be two VCSs in X such that $\min\{\max\left\{A_{V}^{+}\left(x\right),B_{V}^{-}\left(x\right)\right\},\max\left\{A_{V}^{-}\left(x\right),B_{V}^{+}\left(x\right)\right\}\}=\left(\lambda \wedge \nu \right)\left(x\right)$$=\max\{\min\left\{A_{V}^{+}\left(x\right),B_{V}^{-}\left(x\right)\right\},\min\left\{A_{V}^{-}\left(x\right),B_{V}^{+}\left(x\right)\right\}\}$ for all x∈X. Then P-intersection $A^{V}$ and $B^{V}$ is an ECVS and an ICVS in X*

**Proof.**  For each x∈X, substitute

$$\psi_{\left(x\right)}:=\min\left\{\max\left\{A_{V}^{+}\left(x\right),B_{V}^{-}\left(x\right)\right\},\max\left\{A_{V}^{-}\left(x\right),B_{V}^{+}\left(x\right)\right\}\right\}$$

and

$$\phi_{\left(x\right)}:=\max\left\{\min\left\{A_{V}^{+}\left(x\right),B_{V}^{-}\left(x\right)\right\},\min\left\{A_{V}^{-}\left(x\right),B_{V}^{+}\left(x\right)\right\}\right\}$$

Then $\psi_{\left(x\right)}$ which is one of the $A_{V}^{-}\left(x\right),B_{V}^{-}\left(x\right),A_{V}^{+}\left(x\right)$ and $B_{V}^{+}\left(x\right)$. We take $\psi_{\left(x\right)}=A_{V}^{-}\left(x\right)$ or $\psi_{\left(x\right)}=A_{V}^{+}\left(x\right)$ only.

If $\psi_{\left(x\right)}=A_{V}^{-}\left(x\right)$, thus

$$B_{V}^{-}\left(x\right)\leq B_{V}^{+}\left(x\right)\leq A_{V}^{-}\left(x\right)\leq A_{V}^{+}\left(x\right)$$

and so $\phi_{\left(x\right)}=B_{V}^{+}\left(x\right)$. This implies that $A_{V}^{-}\left(x\right)=\psi_{\left(x\right)}=\left(\lambda \wedge \nu \right)\left(x\right)=\phi_{\left(x\right)}=B_{V}^{+}\left(x\right)$. Thus,

$$B_{V}^{-}\left(x\right)\leq B_{V}^{+}\left(x\right)=\left(\lambda \wedge \nu \right)\left(x\right)=A_{V}^{-}\left(x\right)\leq A_{V}^{+}\left(x\right).$$

implies that $\left(\lambda \wedge \nu \right)\left(x\right)=B_{V}^{+}\left(x\right)={\left(A\cap B\right)}^{+}\left(x\right)$. Thus,

$$\left(\lambda \wedge \nu \right)\left(x\right)\notin \left({\left(A\cap B\right)}^{-}\left(x\right),{\left(A\cap B\right)}^{+}\left(x\right)\right)$$

and ${\left(A\cap B\right)}^{-}\left(x\right)\leq \left(\lambda \wedge \nu \right)\left(x\right)\leq {\left(A\cap B\right)}^{+}\left(x\right)$.

If $\psi_{\left(x\right)}=A_{V}^{+}\left(x\right)$, thus, $B_{V}^{-}\left(x\right)\leq A_{V}^{+}\left(x\right)\leq B_{V}^{+}\left(x\right)$ and so $\left(\lambda \wedge \nu \right)\left(x\right)=A_{V}^{+}\left(x\right)={\left(A\cap B\right)}^{+}\left(x\right)$. Then $\left(\lambda \wedge \nu \right)\left(x\right)\notin \left({\left(A\cap B\right)}^{-}\left(x\right),{\left(A\cap B\right)}^{+}\left(x\right)\right)$ and ${\left(A\cap B\right)}^{-}\left(x\right)\leq \left(\lambda \wedge \nu \right)\left(x\right)\leq {\left(A\cap B\right)}^{+}\left(x\right)$. Therefore, the P-intersection of $A^{V}$ and $B^{V}$ is an ECVS and ICVS in *X*. □

We provide the condition of a P-union of two ECVSs to become an ECVS.

**Theorem** **12.** *Let $A^{V}=\langle A_{V}\left(x\right),\lambda_{V}\left(x\right)\rangle$ and $B^{V}=\langle B_{V}\left(x\right),\nu_{V}\left(x\right)\rangle$ be two EVCSs in X such that $\min\{\max\left\{A_{V}^{+}\left(x\right),B_{V}^{-}\left(x\right)\right\},\max\left\{A_{V}^{-}\left(x\right),B_{V}^{+}\left(x\right)\right\}\}$ > (λ∧ν)(x)$\geq \max\{\min\left\{A_{V}^{+}\left(x\right),B_{V}^{-}\left(x\right)\right\},\min\left\{A_{V}^{-}\left(x\right),B_{V}^{+}\left(x\right)\right\}\}\forall x\in X$. Then the P-union $A^{V}$ and $B^{V}$ is an ECVS in X.*

**Proof.**  For each x∈X, substitute

$$\psi_{\left(x\right)}:=\min\left\{\max\left\{A_{V}^{+}\left(x\right),B_{V}^{-}\left(x\right)\right\},\max\left\{A_{V}^{-}\left(x\right),B_{V}^{+}\left(x\right)\right\}\right\}$$

and

$$\phi_{\left(x\right)}:=\max\left\{\min\left\{A_{V}^{+}\left(x\right),B_{V}^{-}\left(x\right)\right\},\min\left\{A_{V}^{-}\left(x\right),B_{V}^{+}\left(x\right)\right\}\right\}$$

Then $\psi_{\left(x\right)}$ is one of the $A_{V}^{-}\left(x\right),B_{V}^{-}\left(x\right),A_{V}^{+}\left(x\right)$ and $B_{V}^{+}\left(x\right)$. We consider $\psi_{\left(x\right)}=A_{V}^{-}\left(x\right)$ or $\psi_{\left(x\right)}=A_{V}^{+}\left(x\right)$ only.

If $\psi_{\left(x\right)}=A_{V}^{-}\left(x\right)$, thus,

$$B_{V}^{-}\left(x\right)\leq B_{V}^{+}\left(x\right)\leq A_{V}^{-}\left(x\right)\leq A_{V}^{+}\left(x\right)$$

and so $\phi_{\left(x\right)}=B_{V}^{+}\left(x\right)$. This implies that

$${\left(A,\cup B\right)}^{-}\left(x\right)=A_{V}^{-}\left(x\right)=\psi_{\left(x\right)}>\left(\lambda \wedge \nu \right)\left(x\right),$$

hence,

$$\left(\lambda \vee \nu \right)\left(x\right)\notin \left({\left(A\cup B\right)}^{-}\left(x\right),{\left(A\cup B\right)}^{+}\left(x\right)\right).$$

If $\psi_{\left(x\right)}=A_{V}^{+}\left(x\right)$, thus $B_{V}^{-}\left(x\right)\leq A_{V}^{+}\left(x\right)\leq B_{V}^{+}\left(x\right)$ and so $\phi_{\left(x\right)}=\max\left\{A_{V}^{-}\left(x\right),B_{V}^{-}\left(x\right)\right\}$. Suppose that $\phi_{\left(x\right)}=A_{V}^{-}\left(x\right).$ We have,

$$B_{V}^{-}\left(x\right)\leq A_{V}^{-}\left(x\right)\leq \left(\lambda \vee \nu \right)\left(x\right)<A_{V}^{+}\left(x\right)\leq B_{V}^{+}\left(x\right),$$

and

$$B_{V}^{-}\left(x\right)\leq A_{V}^{-}\left(x\right)<\left(\lambda \vee \nu \right)\left(x\right)<A_{V}^{+}\left(x\right)\leq B_{V}^{+}\left(x\right)$$

or

$$B_{V}^{-}\left(x\right)\leq A_{V}^{-}\left(x\right)=\left(\lambda \vee \nu \right)\left(x\right)\leq A_{V}^{+}\left(x\right)\leq B_{V}^{+}\left(x\right)$$

That is a contradiction for that fact $A^{V}=\langle A_{V}\left(x\right),\lambda_{V}\left(x\right)\rangle$ and $B^{V}=\langle B_{V}\left(x\right),\nu_{V}\left(x\right)\rangle$ are ECVSs in *X* in the first case. For the next case, we will show that $\left(\lambda \vee \nu \right)\left(x\right)\notin \left({\left(A\cup B\right)}^{-}\left(x\right),{\left(A\cup B\right)}^{+}\left(x\right)\right)$ because ${\left(A\cup B\right)}^{-}\left(x\right)=A_{V}^{-}\left(x\right)=\left(\lambda \vee \nu \right)\left(x\right).$ Suppose $\phi_{\left(x\right)}=B_{V}^{-}\left(x\right).$ We have,

$$A_{V}^{-}\left(x\right)\leq B_{V}^{-}\left(x\right)\leq \left(\lambda \vee \nu \right)\left(x\right)\leq A_{V}^{+}\left(x\right)\leq B_{V}^{+}\left(x\right),$$

which means

$$A_{V}^{-}\left(x\right)\leq B_{V}^{-}\left(x\right)<\left(\lambda \vee \nu \right)\left(x\right)<A_{V}^{+}\left(x\right)\leq B_{V}^{+}\left(x\right)$$

or

$$A_{V}^{-}\left(x\right)\leq B_{V}^{-}\left(x\right)=\left(\lambda \vee \nu \right)\left(x\right)<A_{V}^{+}\left(x\right)\leq B_{V}^{+}\left(x\right).$$

It contradicts $A_{V}^{-}\left(x\right)\leq B_{V}^{-}\left(x\right)<\left(\lambda \vee \nu \right)\left(x\right)<A_{V}^{+}\left(x\right)\leq B_{V}^{+}\left(x\right)$, for the fact $A^{V}=\langle A_{V}\left(x\right),\lambda_{V}\left(x\right)\rangle$ and $B^{V}=\langle B_{V}\left(x\right),\nu_{V}\left(x\right)\rangle$ are ECVSs in *X*. In the case

$$A_{V}^{-}\left(x\right)\leq B_{V}^{-}\left(x\right)=\left(\lambda \vee \nu \right)\left(x\right)<A_{V}^{+}\left(x\right)\leq B_{V}^{+}\left(x\right),$$

we get $\left(\lambda \wedge \nu \right)\left(x\right)\notin \left({\left(A\cup B\right)}^{-}\left(x\right),{\left(A\cup B\right)}^{+}\left(x\right)\right)$ since ${\left(A\cup B\right)}^{-}\left(x\right)=B_{V}^{-}\left(x\right)=\left(\lambda \vee \nu \right)\left(x\right)$. Thus, a P-union of $A^{V}$ and $B^{V}$ is an ECVS in *X*. □

**Theorem** **13.** *Let $A^{V}=\langle A_{V}\left(x\right),\lambda_{V}\left(x\right)\rangle$ and $B^{V}=\langle B_{V}\left(x\right),\nu_{V}\left(x\right)\rangle$ be two ECVSs in X. If for each x∈X such that $\min\{\max\left\{A_{V}^{+}\left(x\right),B_{V}^{-}\left(x\right)\right\},\max\left\{A_{V}^{-}\left(x\right),B_{V}^{+}\left(x\right)\right\}\}>\left(\lambda \wedge \nu \right)\left(x\right)$$\geq \max\{\min\left\{A_{V}^{+}\left(x\right),B_{V}^{-}\left(x\right)\right\},\min\left\{A_{V}^{-}\left(x\right),B_{V}^{+}\left(x\right)\right\}\}$, then the R-union $A^{V}$ and $B^{V}$ is an ECVS in X.*

**Proof.**  For each x∈X, substitute

$$\psi_{\left(x\right)}:=\min\left\{\max\left\{A_{V}^{+}\left(x\right),B_{V}^{-}\left(x\right)\right\},\max\left\{A_{V}^{-}\left(x\right),B_{V}^{+}\left(x\right)\right\}\right\}$$

and

$$\phi_{\left(x\right)}:=\max\left\{\min\left\{A_{V}^{+}\left(x\right),B_{V}^{-}\left(x\right)\right\},\min\left\{A_{V}^{-}\left(x\right),B_{V}^{+}\left(x\right)\right\}\right\}.$$

Then $\psi_{\left(x\right)}$ is one of the $A_{V}^{-}\left(x\right),B_{V}^{-}\left(x\right),A_{V}^{+}\left(x\right)$ and $B_{V}^{+}\left(x\right)$. Consider the case of $\psi_{\left(x\right)}=B_{V}^{-}\left(x\right)$ or $\psi_{\left(x\right)}=B_{V}^{+}\left(x\right)$.

If $\psi_{\left(x\right)}=A_{V}^{+}\left(x\right)$, thus,

$$A_{V}^{-}\left(x\right)\leq A_{V}^{+}\left(x\right)\leq B_{V}^{-}\left(x\right)\leq B_{V}^{+}\left(x\right)$$

and $\phi_{\left(x\right)}=A_{V}^{+}\left(x\right)$. Then the first part of inequality

$${\left(A\cup B\right)}^{-}\left(x\right)=B_{V}^{-}\left(x\right)=\psi_{\left(x\right)}>\left(\lambda \wedge \nu \right)\left(x\right)$$

and $\left(\lambda \wedge \nu \right)\left(x\right)\notin \left({\left(A\cup B\right)}^{-}\left(x\right),{\left(A\cup B\right)}^{+}\left(x\right)\right).$

If $\psi_{\left(x\right)}=B_{V}^{+}\left(x\right)$, then

$$A_{V}^{-}\left(x\right)\leq B_{V}^{+}\left(x\right)\leq A_{V}^{+}\left(x\right)$$

and $\phi_{\left(x\right)}=\max\left\{A_{V}^{-}\left(x\right),B_{V}^{-}\left(x\right)\right\}$. Suppose $\phi_{\left(x\right)}=A_{V}^{-}\left(x\right)$. Thus,

$$B_{V}^{-}\left(x\right)\leq A_{V}^{-}\left(x\right)\leq \left(\lambda \wedge \nu \right)\left(x\right)<B_{V}^{+}\left(x\right)\leq A_{V}^{+}\left(x\right)$$

which implies that

$$B_{V}^{-}\left(x\right)\leq A_{V}^{-}\left(x\right)<\left(\lambda \wedge \nu \right)\left(x\right)<B_{V}^{+}\left(x\right)\leq A_{V}^{+}\left(x\right)$$

or

$$B_{V}^{-}\left(x\right)\leq A_{V}^{-}\left(x\right)=\left(\lambda \wedge \nu \right)\left(x\right)\leq B_{V}^{+}\left(x\right)\leq A_{V}^{+}\left(x\right)$$

For the case $B_{V}^{-}\left(x\right)\leq A_{V}^{-}\left(x\right)<\left(\lambda \wedge \nu \right)\left(x\right)<B_{V}^{+}\left(x\right)\leq A_{V}^{+}\left(x\right)$, it contradicts the fact that $A^{V}=\langle A_{V}\left(x\right),\lambda_{V}\left(x\right)\rangle$ and $B^{V}=\langle B_{V}\left(x\right),\nu_{V}\left(x\right)\rangle$ are ECVSs in *X*. For the case

$$B_{V}^{-}\left(x\right)\leq A_{V}^{-}\left(x\right)=\left(\lambda \wedge \nu \right)\left(x\right)\leq B_{V}^{+}\left(x\right)\leq A_{V}^{+}\left(x\right)$$

we have $\left(\lambda \wedge \nu \right)\left(x\right)\notin \left({\left(A\cup B\right)}^{-}\left(x\right),{\left(A\cup B\right)}^{+}\left(x\right)\right)$ since $\left({\left(A\cup B\right)}^{-}\left(x\right)\right)=A_{V}^{-}\left(x\right)=\left(\lambda \wedge \nu \right)\left(x\right)$.

Suppose $\phi_{\left(x\right)}=B_{V}^{-}\left(x\right).$ We have,

$$A_{V}^{-}\left(x\right)\leq B_{V}^{-}\left(x\right)\leq \left(\lambda \wedge \nu \right)\left(x\right)\leq B_{V}^{+}\left(x\right)\leq A_{V}^{+}\left(x\right).$$

Hence,

$$A_{V}^{-}\left(x\right)\leq B_{V}^{-}\left(x\right)<\left(\lambda \wedge \nu \right)\left(x\right)<B_{V}^{+}\left(x\right)\leq A_{V}^{+}\left(x\right)$$

or

$$A_{V}^{-}\left(x\right)\leq B_{V}^{-}\left(x\right)=\left(\lambda \wedge \nu \right)\left(x\right)\leq B_{V}^{+}\left(x\right)\leq A_{V}^{+}\left(x\right).$$

For the case $A_{V}^{-}\left(x\right)\leq B_{V}^{-}\left(x\right)<\left(\lambda \wedge \nu \right)\left(x\right)<B_{V}^{+}\left(x\right)\leq A_{V}^{+}\left(x\right)$, it is a contradiction since $A^{V}=\langle A_{V}\left(x\right),\lambda_{V}\left(x\right)\rangle$ and $B^{V}=\langle B_{V}\left(x\right),\nu_{V}\left(x\right)\rangle$ are ECVSs in *X*. For the case

$$A_{V}^{-}\left(x\right)\leq B_{V}^{-}\left(x\right)=\left(\lambda \wedge \nu \right)\left(x\right)\leq B_{V}^{+}\left(x\right)\leq A_{V}^{+}\left(x\right),$$

we notice that $\left(\lambda \wedge \nu \right)\left(x\right)\notin \left({\left(A\cup B\right)}^{-}\left(x\right),{\left(A\cup B\right)}^{+}\left(x\right)\right)$ since $\left({\left(A\cup B\right)}^{-}\left(x\right)\right)=B_{V}^{-}\left(x\right)=\left(\lambda \wedge \nu \right)\left(x\right)$. Hence the R-union of $A^{V}$ and $B^{V}$ is an ECVS in *X*. □

For the R-intersection we provide the condition of two ECVSs to be an ECVS.

**Theorem** **14.** *Let $A^{V}=\langle A_{V}\left(x\right),\lambda_{V}\left(x\right)\rangle$ and $B^{V}=\langle B_{V}\left(x\right),\nu_{V}\left(x\right)\rangle$ be two ECVSs in X. If for each x∈X such that $\min\{\max\left\{A_{V}^{+}\left(x\right),B_{V}^{-}\left(x\right)\right\},\max\left\{A_{V}^{-}\left(x\right),B_{V}^{+}\left(x\right)\right\}\}\geq \left(\lambda \wedge \nu \right)\left(x\right)$$>\max\{\min\left\{A_{V}^{+}\left(x\right),B_{V}^{-}\left(x\right)\right\},\min\left\{A_{V}^{-}\left(x\right),B_{V}^{+}\left(x\right)\right\}\}$, then the R-intersection of $A^{V}$ and $B^{V}$ is an ECVS in X.*

**Proof.**  The proof is similar to that of Theorem 13. □

For R-intersection we provide the condition of two CVSs to be both an ECVS and ICVS.

**Theorem** **15.** *Let $A^{V}=\langle A_{V}\left(x\right),\lambda_{V}\left(x\right)\rangle$ and $B^{V}=\langle B_{V}\left(x\right),\nu_{V}\left(x\right)\rangle$ be CVSs in X. If for each x∈X such that $\min\{\max\left\{A_{V}^{+}\left(x\right),B_{V}^{-}\left(x\right)\right\},\max\left\{A_{V}^{-}\left(x\right),B_{V}^{+}\left(x\right)\right\}\}=\left(\lambda \wedge \nu \right)\left(x\right)$$=\max\{\min\left\{A_{V}^{+}\left(x\right),B_{V}^{-}\left(x\right)\right\},\min\left\{A_{V}^{-}\left(x\right),B_{V}^{+}\left(x\right)\right\}\},$∀x∈X, then the R-intersection of $A^{V}$ and $B^{V}$ is an ECVS and an ICVS in X.*

**Proof.**  The proof is similar to that of Theorem 11. □

For the R-union we provide the condition of two ICVSs to be an ECVS.

**Theorem** **16.** *Let $A^{V}=\langle A_{V}\left(x\right),\lambda_{V}\left(x\right)\rangle$ and $B^{V}=\langle B_{V}\left(x\right),\nu_{V}\left(x\right)\rangle$ be ICVSs in X. If $\left(\lambda \wedge \nu \right)\left(x\right)\leq \max\{A_{V}^{-}\left(x\right),B_{V}^{-}\left(x\right)\}$∀x∈X, then the R-union of $A^{V}$ and $B^{V}$ is an ECVS in X.*

**Proof.**  Straightforward. □

For the R-intersection we provide the condition of two ICVSs to be an ECVS.

**Theorem** **17.** *Let $A^{V}=\langle A_{V}\left(x\right),\lambda_{V}\left(x\right)\rangle$ and $B^{V}=\langle B_{V}\left(x\right),\nu_{V}\left(x\right)\rangle$ be ICVSs in X. If $\left(\lambda \vee \nu \right)\left(x\right)\leq \min\{A_{V}^{+}\left(x\right),B_{V}^{+}\left(x\right)\}$ for all x∈X, then the R-intersection of $A^{V}$ and $B^{V}$ is an ECVS in X.*

**Proof.**  Straightforward. □

For the R-union we provide the condition of two ECVSs to be an ICVS.

**Theorem** **18.** *Let $A^{V}=\langle A_{V}\left(x\right),\lambda_{V}\left(x\right)\rangle$ and $B^{V}=\langle B_{V}\left(x\right),\nu_{V}\left(x\right)\rangle$ be ICVSs in X such that $\min\left\{\max\left\{A_{V}^{+}\left(x\right),B_{V}^{-}\left(x\right)\right\},\max\left\{A_{V}^{-}\left(x\right),B_{V}^{+}\left(x\right)\right\}\right\}\leq \left(\lambda \vee \nu \right)\left(x\right)\leq \max\left\{A_{V}^{+}\left(x\right),B_{V}^{+}\left(x\right)\right\}$ for all x∈X, then the R-union of $A^{V}$ and $B^{V}$ is an ICVS in X.*

**Proof.**  Straightforward. □

## 4. Similarity Measure of Cubic Vague Sets

The most important mathematical tool for solving problems in pattern recognition and clustering analysis is similarity measure. Therefore, in this section we will propose the similarity measures between two CVSs, which will then be applied to a pattern recognition problem.

**Definition** **13.** *A real valued function $S:C_{V}^{X}$→[0,1] is a similarity measure between two CVSs $A_{1}^{V}$ and $A_{2}^{V}$ if S satisfies all of the following axioms:*

(*S*1)  $0\leq |S(A_{1}^{V},A_{1}^{V})|\leq 1$;
(*S*2)  $S\left(A_{1}^{V},A_{2}^{V}\right)=S\left(A_{2}^{V},A_{1}^{V}\right)),$;
(*S*3)  $S(A_{1}^{V},A_{2}^{V})=1$⟺$A_{1}^{V}=A_{2}^{V}$
(*S*4)  $\forall A_{1}^{V},A_{2}^{V}$ and $A_{3}^{V}\in C_{V}^{X}$, if $A_{1}^{V}\subseteq A_{2}^{V}\subseteq A_{3}^{V}$, then $S\left(A_{1}^{V},A_{3}^{V}\right)\leq S\left(A_{1}^{V},A_{2}^{V}\right)$ and $S\left(A_{1}^{V},A_{3}^{V}\right)\leq S\left(A_{2}^{V},A_{3}^{V}\right)$

Next, we give the similarity measurement between two CVSs.

**Definition** **14.** *Let $X=\{x_{1},x_{2},x_{3}\}$ be the universe of the objects, $A_{1}^{V}=\langle A_{V}^{1},\lambda_{V}^{1}\rangle$ and $A_{2}^{V}=\langle A_{V}^{2},\lambda_{V}^{2}\rangle$ are two families of cubic vague sets in X. The similarity measurement between $A_{1}^{V}$ and $A_{2}^{V}$ is defined by the function $S(A_{1}^{V},A_{2}^{V})$, where* $S\left(A_{1}^{V},A_{2}^{V}\right)=1-\frac{1}{6n}\sum_{i=1}^{n}\left(|t_{A_{V}^{1}}^{-}\left(x_{i}\right)-t_{A_{V}^{2}}^{-}\left(x_{i}\right)-\left[f_{A_{V}^{1}}^{-}\left(x_{i}\right)-f_{A_{V}^{2}}^{-}\left(x_{i}\right)\right]\left|+\right|t_{A_{V}^{1}}^{+}\left(x_{i}\right)-t_{A_{V}^{2}}^{+}\left(x_{i}\right)-\left[f_{A_{V}^{1}}^{+}\left(x_{i}\right)-f_{A_{V}^{2}}^{+}\left(x_{i}\right)\right]\left|+\right|t_{\lambda_{V}^{1}}\left(x_{i}\right)-t_{\lambda_{V}^{2}}\left(x_{i}\right)-\left[f_{\lambda_{V}^{1}}\left(x_{i}\right)-f_{\lambda_{V}^{2}}\left(x_{i}\right)\right]|\right)$

### Application of the Similarity Measurement Method in a Pattern Recognition Problem

The measures of fuzzy sets and their hybrid methods help us to solve problems in many real-life areas, especially in the field of pattern recognition and image processing, among others. In this section, we will examine the similarity measures of two CVSs of a pattern recognition problem. We construct an algorithm, and suppose that $S(A^{V},A_{i}^{V})\geq 0.6$ is the ideal pattern.

*Step 1.*  Firstly, we construct an ideal VCS $A^{V}=\langle A_{V},\lambda_{V}\rangle$.
*Step 2.*  Then, we construct cubic vague sets $A_{j}^{V}=\langle A_{V}^{j},\lambda_{V}^{j}\rangle$, j=1,2,...,k, on *X* for a sample patterns which are under consideration.
*Step 3.*  The similarity measures between the sample patterns $A_{j}^{V}=\langle A_{V}^{j},\lambda_{V}^{j}\rangle$, j=1,2,...,k and ideal pattern $A^{V}=\langle A_{V},\lambda_{V}\rangle$ are calculated using the formula given in Definition 14.
*Step 4.*  If $S(A^{V},A_{j}^{V})\leq 0.6$ then the pattern $A_{j}^{V}$ is to be recognized to belong to the ideal Pattern $A^{V}$ and if $S(A^{V},A_{j}^{V})>0.6$ then the pattern $A_{j}^{V}$ is not to be recognized for an ideal Pattern $A^{V}$.

Now, we provide a numerical application example for similarity measurement of two CVSs in a pattern recognition problem. Through the example, we will illustrate which one of the sample patterns belongs to the ideal pattern.

**Example** **9.** *Consider a simple pattern recognition problem involving three sample patterns and an ideal pattern. The objective of the problem is to determine which one of the three sample patterns belongs to the ideal pattern. Let $X=\{x_{1},x_{2},x_{3}\}$ be the universe. Three patterns denoted as pattern 1, pattern 2 and pattern 3 are the designated sample patterns, whereas pattern 4 is the designated ideal pattern. These three patterns are modeled using the CVS model, with the CVSs $A_{1}^{V}$; $A_{2}^{V}$; $A_{3}^{V}$ and $A^{V}$ representing the information and data of patterns 1, 2, 3 and 4, respectively.*

*[Step 1.] Construct an ideal CVS $A^{V}=\langle A_{V},\lambda_{V}\rangle$ on X as;*

$A^{V}=\langle \left\{\frac{\langle [0.3,0.3],[0.7,0.7]\rangle }{x_{1}^{+}},\frac{\langle [0.4,0.4],[0.5,0.6]\rangle }{x_{2}^{+}},\frac{\langle [0.1,0.1],[0.8,0.9]\rangle }{x_{3}^{+}}\right\},\left\{\frac{\left(0.8,0.8\right)}{x_{1}},\frac{\left(0.6,0.6\right)}{x_{2}},\frac{\left(0.9,1\right)}{x_{3}}\right\}\rangle .$

*[Step 2.] Construct CVSs $A_{j}^{V}=\langle A_{V}^{j},\lambda_{V}^{j}\rangle$, j=1,2,3 on X for the sample patterns as;*

$A_{1}^{V}=\langle \left\{\frac{\langle \left[0.1,0.3\right],\left[0.3,0.3\right]\rangle }{x_{1}^{+}},\frac{\langle \left[0.0,0.2\right],\left[0.2,0.4\right]\rangle }{x_{2}^{+}},\frac{\langle \left[0.0,0.1\right],\left[0.1,0.1\right]\rangle }{x_{3}^{+}}\right\},\left\{\frac{\left(0.1,0.1\right)}{x_{1}},\frac{\left(0.1,0.1\right)}{x_{2}}\frac{\left(0,0.1\right)}{x_{3}}\right\}\rangle$

$A_{2}^{V}=\langle \left\{\frac{\langle \left[0.4,0.4\right],\left[0.5,0.6\right]\rangle }{x_{1}^{+}},\frac{\langle \left[0.1,0.2\right],\left[0.1,0.7\right]\rangle }{x_{2}^{+}},\frac{\langle \left[0.2,0.4\right],\left[0.4,0.5\right]\rangle }{x_{3}^{+}}\right\},\left\{\frac{\left(0.1,0.8\right)}{x_{1}},\frac{\left(0.7,0.7\right)}{x_{2}}\frac{\left(0.5,1\right)}{x_{3}}\right\}\rangle$

$A_{3}^{V}=\langle \left\{\frac{\langle \left[0.1,0.2\right],\left[0.1,0.2\right]\rangle }{x_{1}^{+}},\frac{\langle \left[0.1,0.2\right],\left[0.2,0.2\right]\rangle }{x_{2}^{+}},\frac{\langle \left[0.1,0.1\right],\left[0.1,0.3\right]\rangle }{x_{3}^{+}}\right\},\left\{\frac{\left(0.1,0.4\right)}{x_{1}},\frac{\left(0.1,0.2\right)}{x_{2}}\frac{\left(0.0,0.1\right)}{x_{3}}\right\}\rangle$

*[Step 3.] Calculate the degree of similarity S between the three sample patterns $A_{j}^{V}$ and the ideal pattern $A^{V}$, ∀j=1,2,3. then the results obtained are*

$S(A^{V},A_{1}^{V})=0.561$

$S(A^{V},A_{2}^{V})=0.833$

$S(A^{V},A_{3}^{V})=0.572$

*[Step 4.] Since $S(A^{V},A_{1}^{V})\leq 0.6$, $S(A^{V},A_{3}^{V})\leq 0.6$ and $S(A^{V},A_{2}^{V})>0.6$, the sample patterns whose corresponding CVS sets are represented by $A_{1}^{V}$ and $A_{3}^{V}$ are recognized as similar patterns of the family of ideal pattern whose CVS set is represented by $A^{V}$ and the pattern whose CVS is represented by $A_{2}^{V}$ does not belong to the family of ideal pattern $A^{V}$.*

To show the advantage of our proposed method using CVS as compared to that of a cubic set [19], let us consider the decision making problem above. It can be seen that cubic set is unable to describe this problem, since it fails to capture the false membership portion of the data in assessing the alternative in the decision-making process.

Note that the CVS is a generalization of a cubic set by adding the concept of vague set to the definition of cubic set. Thus, as shown in the decision making problem above, the CVS has the ability to handle uncertainties, imprecise and vagueness information considering both the truth-membership and falsity-membership values, whereas cubic set can only handle the uncertainties information without taking into account the truth-membership and falsity-membership values. This indeed illustrates the core advantage of CVS against that of CS.

## 5. Conclusions

A new concept of a cubic set namely the cubic vague set is introduced by incorporating the features of a vague set and a cubic set. Several properties and theorems of cubic vague set are defined and proven involving ECVS or ICVS. We have derived different conditions for different operations of two ICVSs (ECVSs) to be an ICVS (ECVS). We have shown that the proposed set and corresponding algorithm can be applied to a decision making problem containing uncertainties. Our future research is finding ways to apply cubic vague set to groups, rings, numerical analysis [35,36,37] and more real life applications.

## Figures and Tables

**Table 1:** Cubic vague set $A^{V}=<A_{V},\lambda_{V}>$.

*X*	$A_{V}$	$\lambda_{V}$
*a*	$\langle [0.1,0.3],[0.3,0.7]\rangle$	(0.5, 0.7)
*b*	$\langle [0.3,0.4],[0.5,0.6]\rangle$	(0.1, 0.3)

**Table 2:** VCSs $A^{V}$ and $B^{V}$.

*X*	$A_{V}\left(x\right)$	$\lambda_{V}\left(x\right)$	*X*	$B_{V}\left(x\right)$	$\nu_{V}\left(x\right)$
*l*	〈[0.2,0.5],[0.5,0.5]〉	(0.1, 0.7)	*l*	〈[0.8,0.9],[0.8,0.8]〉	(0.9, 0.9)
*m*	〈[0.3,0.4],[0.3,0.3]〉	(0.7, 0.8)	*m*	〈[0.3,0.4],[0.5,0.6]〉	(0.8, 0.9)

**Table 3:** VCSs $A^{V}$ and $B^{V}$.

*X*	$A_{V}\left(x\right)$	$\lambda_{V}\left(x\right)$	*X*	$B_{V}\left(x\right)$	$\nu_{V}\left(x\right)$
*k*	〈[0.3,0.5],[0.2,0.2]〉	(0.5, 0.7)	*k*	〈[0.6,0.7],[0.4,0.7]〉	(0.35, 0.45)
*l*	〈[0.2,0.4],[0.1,0.1]〉	(0.1, 0.5)	*l*	〈[0,0.6],[0.7,0.8]〉	(0.3, 0.35)
*m*	〈[0.1,0.1],[0.1,0.3]〉	(0.4, 0.6)	*m*	〈[0.1,0.1],[0.7,0.8]〉	(0.2, 0.9)

**Table 4:** CVSs $A^{V}$ and $B^{V}$.

*X*	$A_{V}\left(x\right)$	$\lambda_{V}\left(x\right)$	*X*	$B_{V}\left(x\right)$	$\nu_{V}\left(x\right)$
*l*	〈[0.2,0.4],[0.4,0.5]〉	(0.6, 0.7)	*l*	〈[0.6,0.8],[0.7,0.8]〉	(0.2, 0.3)
*m*	〈[0.3,0.3],[0.4,0.5]〉	(0.2, 0.2)	*m*	〈[0.2,0.2],[0.1,0.3]〉	(0.4, 0.5)
